# ﻿Phylogeny, morphology and chemistry reveal two new multispored species in the *Lecanorasubfusca* group (Lecanoraceae, Ascomycota)

**DOI:** 10.3897/mycokeys.99.108462

**Published:** 2023-08-07

**Authors:** Lijuan Li, Yanyun Zhang, Christian Printzen

**Affiliations:** 1 Goethe University Frankfurt, 60438, Frankfurt am Main, Germany Goethe University Frankfurt Frankfurt am Main Germany; 2 Senckenberg Research Institute and Natural History Museum, 60325, Frankfurt am Main, Germany Senckenberg Research Institute and Natural History Museum Frankfurt am Main Germany; 3 College of Life Sciences, Anhui Normal University, 241000, Wuhu, China Anhui Normal University Wuhu China

**Keywords:** Ascospores, China, identification key, *Lecanora*, lichen, taxonomy

## Abstract

Two new multispored species from China, *Lecanoraanhuiensis* Li J. Li & Printzen, **sp. nov.** and *Lecanorapseudojaponica* Li J. Li & Printzen, **sp. nov.** are described and illustrated here, based on morphological, chemical and molecular evidence. *Lecanoraanhuiensis* is characterised by an epruinose, yellowish-brown to deep brown apothecial disc, an epihymenium with fine crystals, an amphithecium with small crystals, 16-spored asci and the presence of zeorin, in addition to atranorin. *Lecanorapseudojaponica* is characterised by an epruinose, red-brown apothecial disc, an epihymenium without crystals, an amphithecium with small crystals, 8 or 16- spored asci and the presence of zeorin and the stictic acid complex, in addition to atranorin. Phylogenetic reconstructions, based on mtSSU, nrITS and nrLSU suggest that these two species are members of the *Lecanorasubfusca* group. They are compared with morphologically similar and phylogenetically related species, based on a nrITS dataset. Phylogenetic results show that the multispored taxa of *Lecanora* are polyphyletic. The number of ascospores per ascus appears to be a taxonomic character of minor importance. Detailed descriptions, discussions and figures for the two new species from China and a key for the multispored species of *Lecanora* worldwide are provided.

## ﻿Introduction

*Lecanora* Ach. is one of the largest genera of lichens, including species with lecanorine apothecia, *Lecanora*-type asci and simple, hyaline ascospores. The majority of species produce eight ascospores per ascus. Multispored species with more than eight spores per ascus are relatively rare amongst *Lecanora*. To date, only 14 species have been reported worldwide: *L.cateilea* (Ach.) A. Massal., *L.bruneri* Imshaug & Brodo, *L.loekoesii* L. Lü, Y. Joshi & Hur, *L.moniliformis* L. Qiu & L. Lü, *L.pleospora* Müll. Arg., *L.praesistens* Nyl., *L.weii* L.F. Han & S.Y. Guo, *L.japonica* Müll. Arg., *L.subjaponica* L. Lü & H.Y. Wang, *L.subpraesistens* Nayaka, Upreti & Lumbsch, *L.shangrilaensis* Z.T. Zhao & L. Lü, *L.strobilinoides* Giralt & Gómez-Bolea, *L.polysphaeridia* Alstrup and *L.sambuci* (Pers.) Nyl. [= *Polyozosiasambuci* (Pers.) S.Y.Kondr., Lőkös & Farkas] (Miyawaki 1988; Alstrup 1993; Guderley and Lumbsch 1999; Nayaka et al. 2006; Han et al. 2009; Lü et al. 2011, 2012; Lü and Zhao 2017; Brodo et al. 2019; Qiu and Lü 2022).

As a result of systematic revisions and phylogenetic studies, several genera or species groups have been segregated from or within *Lecanora* sensu lato (s.lat.) (Kalb 1991; Rodriguez-Flakus and Printzen 2014; Zhao et al. 2016; Kondratyuk et al. 2019; Davydov et al. 2021). In this context, the attribution of multispored species to these groups has often been discussed, indicating that the multispored species are polyphyletic within *Lecanora* s.lat. However, a more comprehensive overview of the species is lacking so far.

*Lecanoracateilea* and *L.bruneri* were assigned to the *L.albella* group in the broad sense since they have pruinose apothecial discs and a poorly-developed amphithecium cortex (Lumbsch et al. 1997; Brodo et al. 2019). *Lecanorapolysphaeridia* belongs to the *L.fuscescens* group, based on its *Biatora*-type ascus and globose ascospores (Alstrup 1993; Øvstedal et al. 2020). ‘*Lecanora’ sambuci* was combined into *Polyozosia* A. Massal. (= *Myriolecis* Clem., *Lecanoradispersa* group), because it conforms with the general circumscription of the genus having a more or less immersed thallus, small apothecia with brown discs and pale margins and no lichen substances (Laundon 2003a; Śliwa 2007; Zhao et.al 2016; Kondratyuk et al. 2019). *Lecanorastrobilinoides* was identified as a member of the *Lecanoravaria* group on the basis of phenotypic characters and closely related to *L.strobilina* (Giralt and Gómez-Bolea 1991; Laundon and Rodney 2003). Laundon and Rodney (2003), on the other hand, considered *L.strobilinoides* to be a geographical race of *L.strobilina* and suggested a new combination L.strobilinasubsp.strobilinoides (Giralt & Gómez-Bolea) J.R.Laundon, since they only differed by ascospore size and number. Subsequently Pérez-Ortega and Kantvilas (2018) confirmed the species status of the two taxa and their position within the *L.symmicta* group by phylogenetic analysis. *Lecanoraweii* and *L.shangrilaensis* are only known from China so far and their phylogenetic position within *Lecanora* is not mentioned in the original descriptions. It might be assumed that *L.weii* is a member of the *L.albella* group since it has heavily pruinose apothecial discs and produces atranorin (Han et al. 2009), while *L.shangrilaensis* might belong to the *L.varia* group, because of the presence of usnic acid instead of atranorin, the yellowish apothecia with prominent margin and its preferred substrate, pine cones (Laundon 2003a; Laundon and Rodney 2003; Lü and Zhao 2017).

As they produce atranorin and large calcium oxalate crystals in the amphithecium, *L.pleospora*, *L.praesistens* and *L.subpraesistens* have been identified as typical members of the *L.subfusca* group (Brodo 1984; Lumbsch 1994). These species mainly differ by the presence or absence of crystals in the epihymenium and chemical traits (Guderley and Lumbsch 1999; Nayaka et al. 2006). In addition, *L.japonica*, *L.loekoesii*, *L.moniliformis* and *L.subjaponica* have been described as members of the *L.subfusca* group, since they all have small oxalate crystals in the amphithecium and also produce atranorin (Miyawaki 1988; Guderley and Lumbsch 1999; Wang et al. 2007; Lü et al. 2011; Lü and Zhao 2017; Qiu and Lü 2022).

These systematic attributions were almost all based on phenotypical characters because genetic data are lacking for most of the species. In addition to discussing the phylogenetic position of the multispored species, it is also interesting to study whether the number of ascospores per ascus is a useful character for species delimitation. Since *Lecanorajaponica* contains 8-spored and multispored asci, Guderley and Lumbsch (1999) considered that the number of ascospores is of minor taxonomic significance in the *L.subfusca* group (Miyawaki 1988). The same is true for *L.cateilea*, containing both 8-spored and multispored asci and several other taxa with ‘(8–)12 –16’-spored asci (Guderley and Lumbsch 1999; Nayaka et al. 2006; Brodo et al. 2019; Qiu and Lü 2022).

While studying the species diversity of the *L.subfusca* group in China, within the ongoing project ‘Lecanomics’ (https://lecanomics.org), two multispored taxa, consistent with the general circumscription of the *L.subfusca* group, came to our attention. One of them contains both 8-spored and multispored asci. A phylogenetic analysis, based on molecular data from multiple collections, indicated that both taxa are so far undescribed and we describe them below in detail. By including all available molecular data of multispored taxa in *Lecanora* s.lat., we attempted to confirm the phylogenetic affinity of these and the newly-described species to genera or species groups within Lecanoraceae. In addition, we also tested whether the number of ascospores may be considered a distinguishing feature for species (or genera) within Lecanoraceae.

## ﻿Materials and methods

### ﻿Phenotypic studies

The specimens in this study are deposited in Anhui Normal University (AHUB), Herbarium Mycologicum Academiae Sinicae-Lichenes (HMAS-L) and Lichen Herbarium Kunming Institute of Botany (KUN-L).

We took macrophotographs using a Zeiss Axio Zoom V16. External morphological characters were studied on air-dried material under a stereomicroscope (Zeiss Stemi SV11). Anatomical features were studied using a light microscope (Zeiss Axioskop 2 plus) on transverse sections of apothecia and thalli, cut with a freezing microtome (Zeiss HYRAX KS 34) to 16–20 µm thickness and mounted in water or lactophenol cotton blue. Spore measurements are presented in the following way: (minimum–) x̄ - SD – x̄ – x̄ + SD (–maximum), where x̄ is the arithmetic mean and SD is the standard deviation (values were rounded to the nearest 0.5 µm), followed by the number of measurements (n). Crystals in apothecia were observed in polarized light (POL), their solubility was studied in 20% nitric acid (HNO_3_) (N) and 10% potassium hydroxide (KOH) (K), N-sol/K-sol means crystals dissolved, N-insol/K-insol means they did not dissolve.

One of the goals of this study was to investigate whether samples with 8-spored and multispored asci or species that produce both kinds of asci belonged to different species. Therefore, chemical and molecular data were generated from apothecia after verifying the number of ascospores on hand-cut sections.

Spot tests were conducted using K and a saturated aqueous solution of sodium hypochlorite (NaClO) (C). High-performance thin layer chromatography (HPTLC) was performed in solvents A, B′ and C to identify lichen chemical compounds, following standardised methods (Culberson and Kristinsson 1970; Arup et al. 1993).

### ﻿DNA extraction, PCR and sequencing

Apothecia were cleaned with acetone before DNA extraction. DNA was extracted using the GeneOn Plant DNA Extraction Kit (GeneOn BioTech, China) by the magnetic bead method or the Chelex® 100 Resin (Bio-Rad, USA) method following Ferencová et al. (2017). The fungal internal transcribed spacer (ITS) region of the rDNA was amplified via polymerase chain reaction (PCR) using the primers ITS1F (Gardes and Bruns 1993) and ITS4 (White et al. 1990). The large subunit of the nuclear ribosomal DNA (LSU) was amplified using the primers AL2R (Mangold et al. 2008) and LR6 (Vilgalys and Hester 1990) and the mitochondrial small subunit (mtSSU) of ribosomal RNA using the primers 16F and 972R (Li et al. 2023). PCR amplifications were performed in 25 µl volumes Ready-To-Go PCR Beads (GE Healthcare Life Sciences, Little Chalfont, Buckinghamshire, UK) containing 5 µl of DNA extract and 1 µl of each primer. Cycling conditions included initial denaturation at 94 °C for 5 min, followed by 4 cycles of 94 °C for 30 s, 54 °C (53 °C for mtSSU) for 45 s and 72 °C for 60 s, 30 cycles of 94 °C for 30 s, 48 °C for 30 s and 72 °C for 60 s and a final extension at 72 °C for 10 min. The PCR products were visualised on 1% agarose gels and sequenced by Macrogen Europe (Amsterdam, The Netherlands) with the same primers as the original PCR amplifications.

### ﻿Phylogenetic analyses

A mtSSU-nrITS-nrLSU concatenated dataset and an nrITS dataset with *Protoparmeliabadia* (Hoffm.) Hafellner and *P.picea* (Hoffm.) Hafellner as outgroup were used for this study, respectively (Zhao et al. 2016). First, each locus was aligned and analysed separately. Sequences were assembled and edited in Geneious Prime 2021.0.3 (https://www.geneious.com/). Each gene dataset was aligned using the MAFFT v.7 online service (https://mafft.cbrc.jp/alignment/server/index.html) and GUIDANCE2 web server (http://guidance.tau.ac.il/) to remove poorly- or ambiguously-aligned regions with the default parameter settings. Before concatenating the single-gene datasets, these were tested for potential incongruencies using the online version of IQ-TREE (Trifinopoulos et al. 2016, http://iqtree.cibiv.univie.ac.at/) with 1000 ultrafast bootstrap replicates. No well-supported conflict was detected.

A Maximum Likelihood (ML) phylogenetic tree with simultaneous inference of the optimal partitioning scheme and substitution models for each data partition was inferred using IQ-TREE, suggesting five initial partitions (mtSSU, ITS1, 5.8S, ITS2, nrLSU). The best-fit model for each partition was selected according to the Bayesian Information Criterion (BIC) as follows: TPM2u+F+R3 for mtSSU, TIMe+G4 for ITS1, TNe+G4 for 5.8S and ITS2 and TNe+I+G for nrLSU. The Branch support was assessed using both ultrafast bootstrap approximation (UFBoot) (Minh et al. 2013) with 10000 replicates and the Shimodaira-Hasegawa-like approximate likelihood ratio test (SH-aLRT) (Guindon et al. 2010) with 1000 replicates. Nodes with support values of both UFBoot ≥ 95% and SH-aLRT ≥ 80% were considered well-supported (Minh et al. 2013). Bayesian reconstructions of phylogenies were performed with MrBayes 3.2.6 (Ronquist et al. 2012) to infer phylogenetic trees applying the models inferred by IQ-TREE and slightly simplified as: HKY+F+I+G4 for mtSSU, SYM+G4 for ITS1, K2P+G4 for 5.8S and ITS2, K2P+I+G for nrLSU. All model parameters were unlinked amongst partitions and we used the default distributions for priors. Two parallel runs of four Markov chains each were run for 4 million generations, sampling every 1000^th^ generation and the first 25% discarded as burn-in. The average standard deviation of split frequencies had fallen below 0.01 at the end of the analysis. Tracer v.1.7 (Rambaut et al. 2018) was used to assess chain convergence by checking the effective sampling size (ESS > 200) for all model parameters. Bayesian posterior probabilities ≥ 0.95, UFBoot ≥ 95% and SH-aLRT ≥ 80% were visualised on the ML tree.

The nrITS dataset was analysed based on Maximum Likelihood (ML) using IQ-TREE with automated substitution model selection with three partitions (ITS1, 5.8S and ITS2). The best-fit models were selected as TIMe+G4 for ITS1, TIM2e+I+G4 for 5.8S and TNe+G4 for ITS2. Branch support was assessed using both ultrafast bootstrap approximation (UFBoot) with 10000 replicates and the Shimodaira-Hasegawa-like approximate likelihood ratio test (SH-aLRT) with 1000 replicates. UFBoot ≥ 95% and SH-aLRT ≥ 80% are given on the tree and the editing and annotation are in Microsoft PowerPoint.

## ﻿Results and discussion

**Figure 1.**
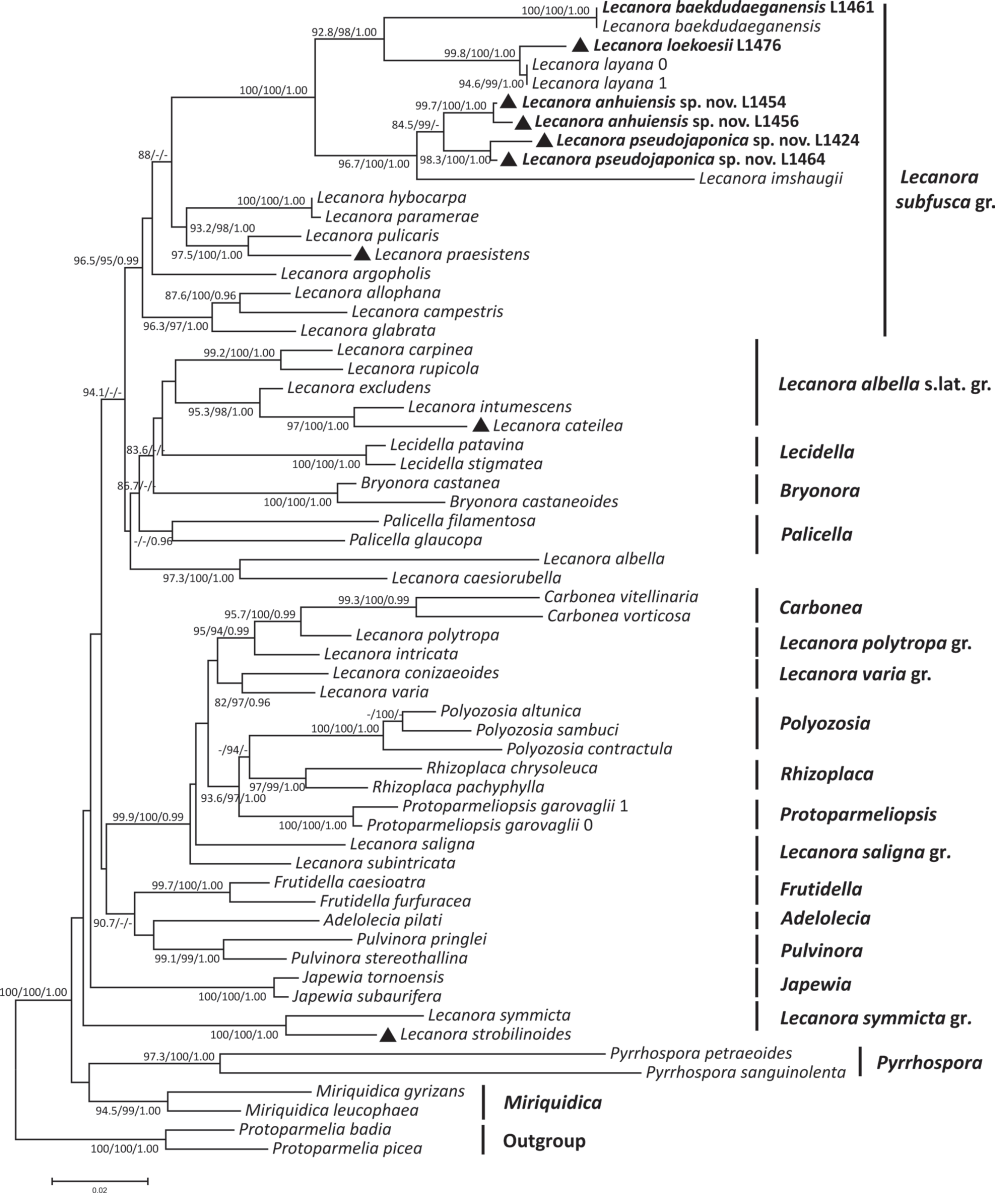
Phylogenetic tree generated from Maximum Likelihood (ML) analysis, based on combined mtSSU, nrITS and nrLSU sequences. SH-aLRT support (%) ≥ 80 / ultrafast bootstrap support (%) ≥ 95 / Bayesian posterior probabilities (PPs) ≥ 0.95 are given above the nodes. Newly-generated sequences are indicated in bold. Multispored species are indicated by black triangles.

**Figure 2.**
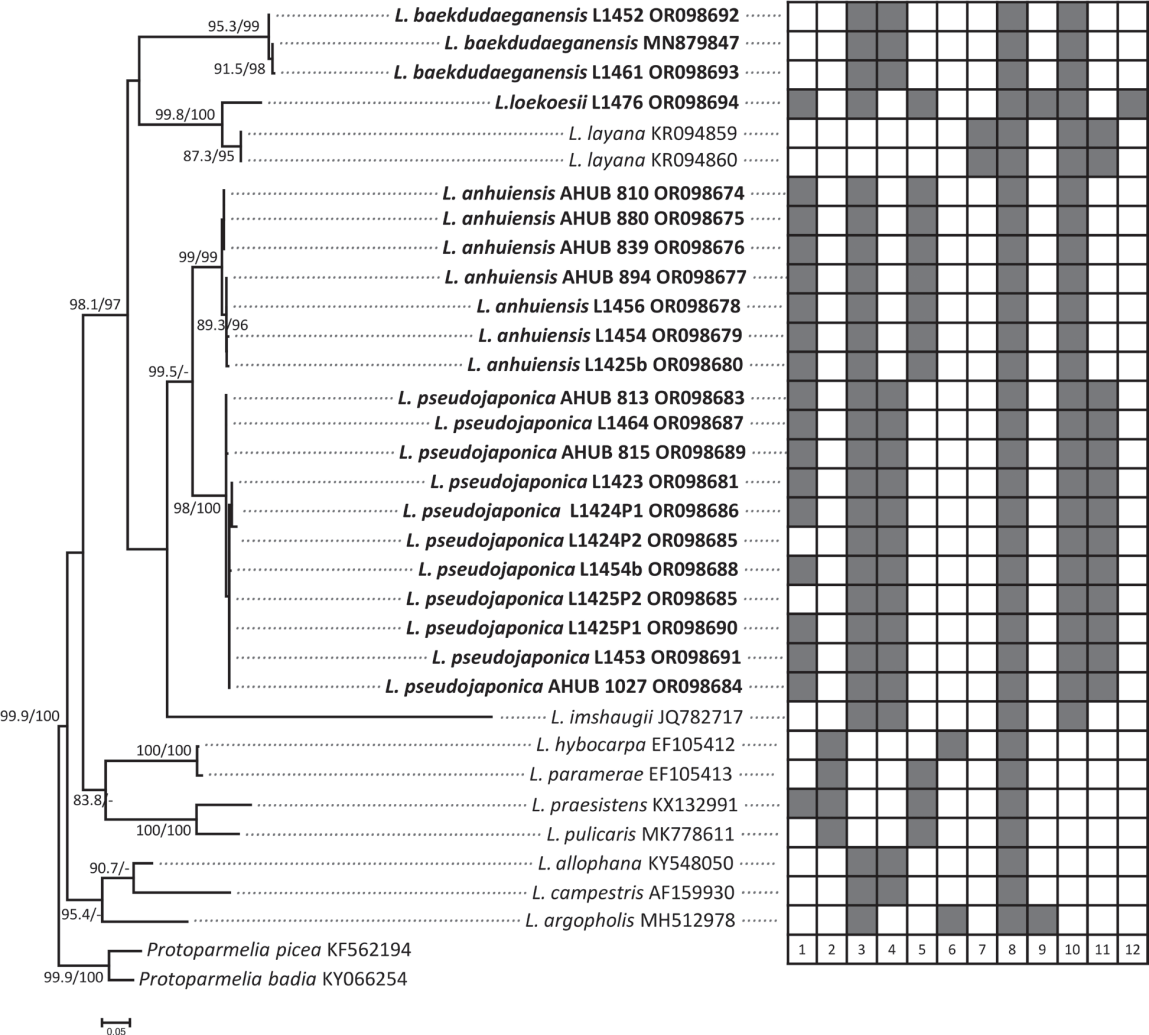
Phylogenetic tree generated from Maximum Likelihood (ML) analysis of the *Lecanorasubfusca* group, emphasising multispored taxa, based on nrITS sequences. SH-aLRT support (%) ≥ 80 / ultrafast bootstrap support (%) ≥ 95 are given above the nodes. Newly-generated sequences are indicated in bold. Phenotypical characters are mapped next to the tree, solid grey rectangles indicate the presence of corresponding features: 1, multispored asci; 2, apothecial amphithecium with large crystals; 3, amphithecium with small crystals; 4, epihymenium without crystals; 5, epihymenium with fine crystals; 6, epihymenium with coarse crystals; 7, thallus with soredia; 8, atranorin; 9, usnic acid; 10, zeorin; 11, stictic acid; 12, norstictic acid.

The mtSSU-nrITS-nrLSU concatenated alignment comprised 61 terminals (Table 1) and included all multispored species with available sequences, as well as species to which they were attributed in previous publications. It also comprised several other major genera and species groups within Lecanoraceae, to figure out the phylogenetic positions of the multispored taxa. The nrITS alignment comprised 34 terminals, including all sequences from the *L.subfusca* group and outgroup shown in Fig. 1, as well as the newly-generated sequences (Fig. 2). Phenotypical characters are mapped on to the ML tree to highlight the significance of the different numbers of ascospores and other distinguishing characters.

**Table 1.** Specimens used for the phylogenetic analyses with the corresponding voucher information and GenBank Accession numbers for mtSSU, nrITS and nrLSU sequences. Newly-obtained sequences in this study are in bold. Species names are followed by their Lecanomics IDs, referring to the project of “Lecanomics” (https://lecanomics.org). “na” indicates that there is no sequence available.

Species name	Voucher details	Country	GenBank Accession number		
			mtSSU	nrITS	nrLSU
*Adeloleciapilati*	Ekman 3373 (BG)	Austria	AY567713	MG925949	AY300826
*Bryonoracastanea*	Westberg PAD321 (UPS)	Sweden	OM417201	OM423658	OM423613
*B.castaneoides*	Svensson 3156 (UPS)	Sweden	OM417202	OM423659	OM423614
*Carboneavitellinaria*	Svensson 3963	Sweden	MZ468129	MZ474888	na
*C.vorticosa*	Tuerk 43031	Antarctica	na	JN873869	na
*Frutidellacaesioatra*	Andersen 91 (BG)	Norway	AY567765	na	AY756349
*F.furfuracea*	Vondrák 26120 (PRA)	Czech Republic	OQ682951	OQ717391	na
*Japewiasubaurifera*	Spribille & Wagner s.n. (GZU)	USA	na	JN009716	KR017230
*J.tornoensis*	Printzen s.n. (hb. BG)	Canada	HQ660559	HQ650656	na
*Lecanoraalbella*	Malíček 7336 (hb. JM)	Czech Republic	KY502423	KY548048	na
*L.allophana*	Malíček 9626 (hb. JM)	Russia	KY502421	KY548050	na
***L.anhuiensis* L1454**	**Ren 20200748 (HMAS-L)**	**China**	**OR096240**	**OR098679**	**OR096274**
***L.anuiensis* L1456**	**Ren 20200731 (HMAS-L)**	**China**	**OR096242**	**OR098678**	**OR096275**
***L.pseudojaponica* L1424**	**Yao 20200919 (HMAS-L)**	**China**	**OR096248**	**OR098686**	**OR096277**
***L.pseudojaponica* L1464**	**Yao 20200932 (HMAS-L)**	**China**	**OR096246**	**OR098687**	**OR096276**
*L.argopholis*	Printzen 12558 (FR)	Austria	MH520108	MH512978	MW257122
*L.baekdudaeganensis*	B.G.Lee 2019-000065 (BDNA)	South Korea	MN879871	MN879847	na
***L.baekdudaeganensis* L1461**	**Zhang 20200762 (HMAS-L)**	**China**	**OR096239**	**OR098693**	**OR096273**
*L.caesiorubella*	Lumbsch 19094a (F)	USA	JQ782666	JN943722	JN939506
*L.campestris*	Arup & Grube 2000 (hb. Arup)	Sweden	na	AF159930	na
*L.carpinea*	Kondratyuk 21337 (KW)	Ukraine	MK693683	MK672827	na
*L.cateilea*	Goward & Poelt (GZU)	Canada	na	AY541250	na
*L.conizaeoides*	Palice 21292 (PRA)	Czech Republic	MT939177	MT938947	na
*L.excludens*	Palice 21929 (PRA)	Norway	MK541649	MK541647	na
*L.glabrata*	Arup L011003 (LD)	Sweden	DQ787360	na	DQ787359
*L.hybocarpa*	Lumbsch s.n. (F)	Spain	EF105417	EF105412	EF105421
*L.imshaugii*	Lumbsch 19273b (F)	USA	JQ782681	JQ782717	na
*L.intricata*	Flakus 29565b (KRAM)	Bolivia	OL604112	OL604030	OL663890
*L.intumescens*	Malíček 8480 (hb. JM)	Czech Republic	KY502441	KY548040	na
*L.layana* 0	Lendemer 37519 (NY)	USA	KR094857	KR094859	na
*L.layana* 1	Lendemer 38131 (NY)	USA	KR094858	KR094860	na
***L.loekoesii* L1476**	**Wei et al. HLJ201400311 (HMAS-L)**	**China**	**OR096237**	**OR098694**	**na**
*L.paramerae*	Lumbsch s.n. (F)	Spain	EF105418	EF105413	EF105422
*L.polytropa*	Flakus 29524 (KRAM)	Bolivia	OL604125	OL604045	OL663904
*L.praesistens*	LIFU083-16 (WSL)	Switzerland	na	KX132991	na
*L.pulicaris*	Malíček 10262 (hb. JM)	Russia	MK778539	MK778611	na
*L.rupicola*	Flakus 29527(KRAM)	Bolivia	OL604094	OL604012	OL663876
*L.saligna*	Palice 21284 (PRA)	Czech Republic	MT939225	MT938996	na
*L.strobilinoides*	Garrido-Benavent s.n. (MA)	Spain	na	MG973238	na
*L.subintricata*	Printzen 15562 (FR)	Japan	MT939239	MT939010	na
*L.symmicta*	Davydov 18083 (hb. Davydov)	Russia	ON553202	ON553209	na
*L.varia*	Kondratyuk 21325 (KW)	Ukraine	MK693694	MK672852	na
*Lecidellapatavina*	ZX 20140501-2	China	KT453845	KT453767	KT453799
*L.stigmatea*	ZX 20141254	China	KT453852	KT453758	KT453808
*Miriquidicagyrizans*	Fryday 10175 (MSC)	USA	MN508282	MN483126	MN460217
*M.leucophaea*	Kossowska 1354 (hb. Kossowska)	Thailand	KP822516	KP822311	KP796397
*Palicella_ filamentosa*	Hauck s. n. (FH)	Germany	HQ660568	HQ650663	HQ660543
*P.glaucopa*	Flakus 2539 (FR)	Argentina	KJ152471	KJ152486	KJ152460
*Polyozosiaaltunica*	Xahidin 20071910 (XJU)	China	MH698407	MH698406	MH698407
*P.contractula*	Brodo 31501(DUKE)	USA	DQ986898	HQ650604	DQ986746
*P.sambuci*	BIOUG24047-E06	Canada	na	KT695378	na
*Protoparmeliabadia*	Fryday 8575	USA	KY012807	KY066254	KY066280
*P.picea*	Haugan 9612 (O)	Norway	na	KF562194	KF562186
*Protoparmeliopsisgarovaglii* 0	Wang et al. 19-63467 (KUN-L)	China	ON807176	ON807160	na
*P.garovaglii* 1	Leavitt 089 (BRY-C)	USA	KT453818	KT453728	KT453775
*Pulvinorapringlei*	McCune 36799 (OSC & ALTB)	USA	MW257153	MW257114	MW257114
*P.stereothallina*	Davydov 14817 (LE & ALTB)	Russia	MW257159	MW257118	MW257118
*Pyrrhosporapetraeoides*	Elix 36816 (F)	EU075531	EU075531	EU075545	EU075521
*P.sanguinolenta*	Elix 28835 (F)	Australia	EU075534	EU075548	EU075523
*Rhizoplacachrysoleuca*	BRY 55000	USA	KT453856	HM577233	KT453812
*R.pachyphylla*	Wang et al. 18-59561 (KUN-L)	China	MN192154	MK778050	na

The three-loci phylogenetic tree (Fig. 1) shows that the species of the *L.subfusca* group form a well-supported monophyletic clade (SH-aLRT = 96.5%, UFBoot = 95%, PP = 0.99), within which two well-supported branches correspond to the two new multispored species, *L.anhuiensis* (SH-aLRT = 99.7%, UFBoot = 100%, PP = 1.00) and *L.pseudojaponica* (SH-aLRT = 98.3%, UFBoot = 100%, PP = 1.00). The two new species show a sister group relationship (SH-aLRT = 84.5%, UFBoot = 99%), both sharing the characteristics of multispored asci, small POL+ crystals in the amphithecium, the major production of atranorin and zeorin, characters in agreement with typical members of the *L.subfusca* group. *Lecanoraimshaugii* with 8-spored asci, reported from Eastern Asian and eastern North America (Miyawaki 1988, 1994), is the most closely-related species (SH-aLRT = 96.7%, UFBoot = 100%, PP = 1.00). Another multispored species, *L.loekoesii*, is closely related to the sorediate *L.layana*. These two species form a group with *L.baekdudaeganensis* reported from South Korea. All these species form a strongly supported clade (SH-aLRT = 100%, UFBoot = 100%, PP = 1.00) nested within the *L.subfusca* group. The multispored *L.praesistens* also belongs to the *L.subfusca* group and appears to be closely related to *L.pulicaris* (SH-aLRT = 97.5%, UFBoot = 100%, PP = 1.00), with which it shares red-brown apothecial discs and large crystals in the amphithecium, but differs by its multispored asci and an epihymenium with coarse crystals (Guderley and Lumbsch 1999). *Lecanoracateilea* and *L.intumescens* form a strongly supported clade (SH-aLRT = 97%, UFBoot = 100%, PP = 1.00). Together with the closely-related *L.excludens*, they produce zeorin as a constant compound in addition to atranorin. The multispored *L.strobilinoides* clusters with *L.symmicta* (SH-aLRT = 100%, UFBoot = 100%, PP = 1.00), in accordance with previous results showing it to be a member of the *L.symmicta* group with (12–)16(–32)-spored asci (Pérez-Ortega and Kantvilas 2018).

Another major clade (UFBoot = 99.9%, SH-aLRT = 100%, PP = 0.99) combines species belonging to *Carbonea*, the *L.polytropa*-, *L.saligna*- and *L.varia* groups, *Polyozosia*, *Protoparmeliopsis* and *Rhizoplaca*. These genera and species groups conform largely to the ‘MPRPS’ clade (Medeiros et al. 2021) which, however, also comprised *Bryonora*, *Carbonea* and the *L.varia* group, but were not included in the analysis of Medeiros et al. (2021). The multispored *Polyozosiasambuci* forms a clade with *P.altunica* and *P.contractula* (SH-aLRT = 100%, UFBoot = 100%, PP = 1.00).

The majority of multispored *Lecanora* species has previously been classified into different genera or species groups, based on phenotypic characters. Our results provide phylogenetic evidence for these assignments. The affinities of multispored taxa in *Lecanora* s.lat. are primarily supported by other phenotypical characters, such as epihymenium and amphithecium characteristics, as well as chemical compounds, rather than the numbers of ascospores.

In the nrITS tree (Fig. 2), the multiple sequences of the two new species, *L.anhuiensis* and *L.pseudojaponica*, formed well-supported clades, respectively. The overall topology of other related species in the tree is consistent with our concatenated tree. Significant phenotypical characters for the species are depicted next to the phylogenetic tree. In the new species *L.pseudojaponica*, we observed the occurrence of apothecia containing both 8-spored and multispored asci growing intermixed on the same thallus (samples L1424 and L1425). We sequenced these apothecia separately (labelled as P1 = multispored and P2 = 8-spored). The results revealed that both types of apothecia had identical sequences where they overlapped (616 bp). This is further support that the number of ascospores is of minor taxonomic significance within the *L.subfusca* group (Guderley and Lumbsch 1999).

### ﻿Taxonomy

#### ﻿ Lecanora anhuiensis

Taxon classificationFungiLecanoralesLecanoraceae

Lijuan Li & Printzen sp. nov.

9E387327-D7B8-5EB6-8AFA-C348A1A78BB3

849250

Fig. 3A–E

##### Diagnosis.

Distinguished from other multispored species of *Lecanora* by brown apothecial discs, fine crystals in the epihymenium, small crystals in the amphithecium and the presence of atranorin and zeorin.

##### Type.

China. Anhui Prov.: Lu’an Ci., Jinzhai Co., The main peak of the Tiantangzhai, Ta-pieh Mountain, 31°06′20″N, 115°46′15″E, alt. 1720 m, on bark, 12 Oct 2020, Ren Qiang 20200731, HMAS-L-0147383—***holotype***.

##### Description.

Thallus corticolous, continuous to rimose to verrucose areolate, thin and tightly attached to the substrate, surface dirty grey to greenish, epruinose, lacking soredia, prothallus not visible.

Apothecia lecanorine, numerous, rounded or deformed by mutual pressure, dispersed to aggregated, sessile to adnate, 0.4–1 mm in diameter; disc plane to slightly concave or convex, yellowish-brown to deep brown, epruinose, margin persistent and prominent, entire or slightly flexuous, cream-white; amphithecium with numerous algal cells, containing small crystals (POL+, K-insol, N-sol); cortex indistinct, interspersed with fine crystals (POL+, K-sol, N-insol); parathecium colourless, 15–25 (–40) µm wide, with fine crystals (POL+, K-sol, N-insol) mostly in the uppermost part; epihymenium with fine crystals (POL+, K-sol, N-insol) on the surface and interspersed to upper part of paraphyses and amongst the apical cells, with deep orangish-brown to deep brown amorphous pigmentation, becoming faint dull brown or dissolving in K; hymenium colourless, 80–110 µm high; paraphyses simple to somewhat branched, ca. 1.5–2 µm thick, tips expanded up to 4 µm; hypothecium colorless, composed of anastomosing hyphae; asci clavate, *Lecanora*-type, 55–70 × 15–25 µm, 16-spored; ascospores simple, hyaline, narrowly ellipsoid to ellipsoid or ovoid, occasionally subglobose, (9.0–)11.0–12.0–13.5(–15.0) × (5.0–)5.5–6.0–7.0(–8.0) µm (n = 74), wall ca. 0.5 µm. Pycnidia not found.

**Figure 3.**
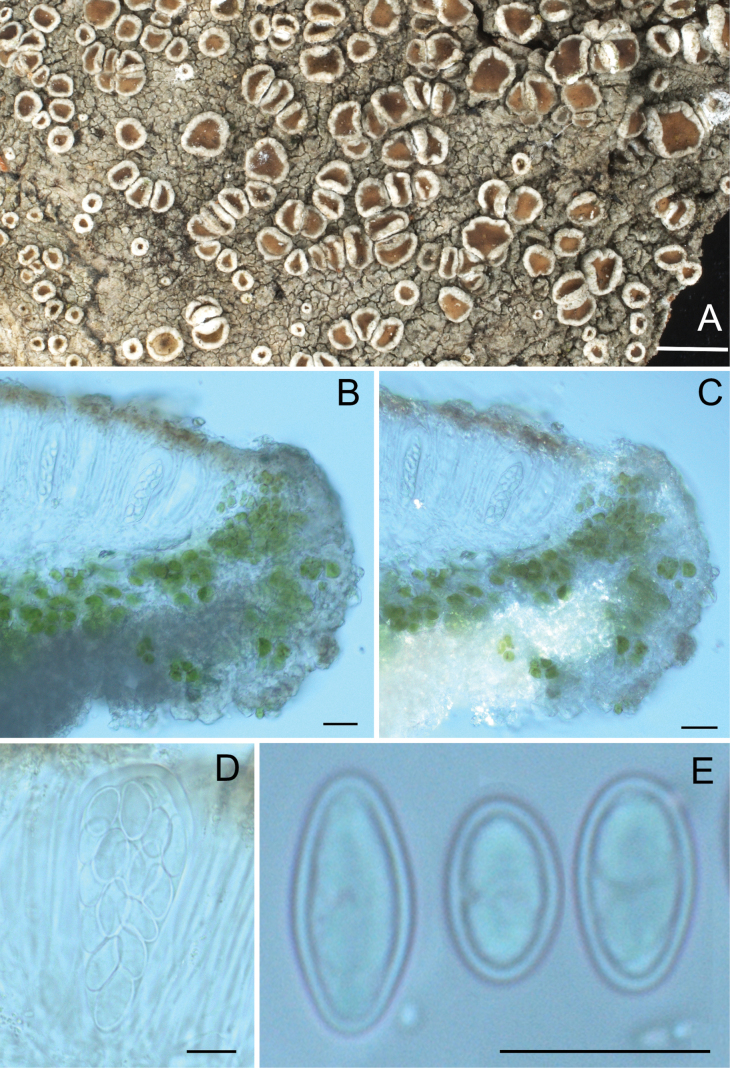
The new species *Lecanoraanhuiensis***A** lichen thallus and apothecia, habit **B** vertical sections of apothecia in normal light **C** vertical sections of apothecia in polarized light **D** 16-spored ascus **E** ascospores. Scale bars: 1 mm (**A**); 20 µm (**B, C**); 10 µm (**D, E**).

##### Chemistry.

Thallus K+ yellow, C-; containing atranorin and zeorin.

##### Distribution.

This species occurs on bark and is known from Anhui Prov., in the south-eastern part of the Ta-pieh Mountains at elevations between 850 and 1720 m. The Ta-pieh Mountains are located at the junction between Anhui, Hubei and Henan Provinces in China.

##### Etymology.

The species is named after its locality in Anhui Province, China.

##### Notes.

*Lecanoraloekoesii* is similar to *L.anhuiensis* in having somewhat yellowish-brown apothecial discs, a granulose epihymenium and small crystals in the amphithcium, but differs in having relatively larger ascospores (12.1–)12.6–15.3(–16.2) × (7–)7.5–8.5(–9) µm in size and producing usnic and norstictic acid in addition to atranorin and zeorin [according to Lü et al. (2011)]. In the species delimitation of the *L.subfusca* group, the type of epihymenial crystals is one of the most important diagnostic characters, as illustrated by Brodo (1984). The original description mentioned that the epihymeniun of *L.loekoesii* contains fine crystals. Subsequently, Wang et al. (2013) examined the holotype and 68 Chinese specimens, suggesting that the crystals are, in fact, coarse. Both types can be distinguished by examining their solubility in N: fine crystals are insoluble in N, while coarse crystals dissolve in N (Brodo 1984). We found that the epihymenial crystals are insoluble in N, indicating the presence of fine crystals, consistent with Lü et al. (2011) and Qiu and Lü (2022).

*Lecanorashangrilaensis*, with yellow to yellowish-brown apothecial discs, a granulose epihymenium and small crystals in the amphithecium, might also be confused with *L.anhuiensis*. However, it can be easily distinguished by the presence of coarse epihymenial crystals, K-soluble crystals in the amphithecium and the production of usnic acid instead of atranorin. *Lecanoraweii* is also similar to *L.anhuiensis* in forming a granulose epihymenium, an amphithecium with K-insoluble small crystals and the presence of atranorin and zeorin, but differs in having heavily pruinose apothecial discs, an epihymenium with coarse crystals (K-sol, N-sol) and 12–16-spored asci (Han et al. 2009).

##### Additional specimens examined.

China: Anhui Prov.: Lu’an Ci., Jinzhai Co., The main peak of the Tiantangzhai, Ta-pieh Mountain, 31°6′20″N, 115°46′15″E, alt. 1720 m, on bark, 12 Oct 2020, Ren Qiang 20200748a (HMAS-L-0147384); Anqing Ci., Yuexi Co., Yangtianwo, Yaoluoping National Nature Reserve, 31°58′11″N, 116°04′10″E, alt. 1160 m, on bark, 15 Oct 2020, Yao Zongting 20200911b (HMAS-L-0147400); Lu′an Ci., Jinzhai Co., JiangjunYan of the TiantangZhai, 31°12′26″N, 115°76′61″E, alt. 1501 m, on bark of *Rhododendron*, 19 Sep 2022, Zhang Yanyun 22-956 (AHUB-00810); Anqing Ci., Yuexi Co., Yangtianwo, Luchai River, 31°03′94″N, 116°11′38″E, alt. 850 m, on the bark of *Pine*, 20 Sep 2022, Zhang Yanyun 22-985 (AHUB-00839); Anqing Ci., Qianshan Co., Xianren Cave, Tianzhu Mountain World Geopark, 30°74′50″N, 116°45′30″E, alt. 1377 m, on bark, 21 Sep 2022, Zhang Yanyun 22-1026 (AHUB-00880); Anqing Ci., Qianshan Co., Qixing Chi, Tianzhu Mountain World Geopark, 30°74′50″N, 116°46′07″E, alt. 1250 m, on the bark of an oak tree, 21 Sep 2022, Zhang Yanyun 22-1040 (AHUB-00894).

#### ﻿ Lecanora pseudojaponica

Taxon classificationFungiLecanoralesLecanoraceae

Lijuan Li & Printzen sp. nov.

D2BDE72C-404C-5D49-9F03-02E121A6EB86

849251

Fig. 4A–G

##### Diagnosis.

Distinguished from other species of *Lecanora* by the red brown apothecial discs, the 16- and 8-spored asci, an epihymenium without crystals, small crystals in the amphithecium and the presence of atranorin, zeorin and the stictic acid complex.

##### Type.

China, Anhui Prov.: Yuexi Co., Yangtianwo, Yaoluoping National Nature Reserve, 31°58′11″N, 116°4′10″E, alt. 1160 m, on bark, 15 Oct 2020, Yao Zongting 20200919, HMAS-L-0147402—***holotype***.

##### Description.

Thallus corticolous, continuous to rimose, thin and tightly attached to the substrate, surface pale green to dull greenish-grey, epruinose, lacking soredia, prothallus black or not visible.

Apothecia lecanorine, numerous, rounded, dispersed to aggregated, sessile to adnate, 0.3–1 mm in diameter; disc plane or moderately concave, yellowish brown to reddish-brown, weakly shiny, epruinose, margin persistent and prominent, entire, cream white or greyish-white; amphithecium with numerous algal cells, small crystals (POL+, K-insol, N-sol); cortex indistinct, interspersed with fine crystals (POL+, K-sol, N-insol); parathecium colourless, 15–20 µm thick, without crystals (POL-); epihymenium without crystals (POL-), with orangish-brown to deep brown amorphous pigmentation, 10–20 µm high, not altered by K (sometimes becoming slightly more dark brown), orange intensifying in N, occasionally topped by a layer of hyaline gel ca. 5 µm thick; hymenium colourless, 60–100 µm high; paraphyses with few anastomoses, weakly branched, ca. 1.5 µm thick, tips expanded to 4 µm with an orangish-brown cap; hypothecium colourless, composed of anastomosing hyphae; asci clavate to narrowly clavate, *Lecanora*-type, 50–65 × 20–25 µm, 8- and 16-spored; ascospores simple, hyaline, ellipsoid to ovoid, (11.0–)13.0–14.5–16.5(–18) × (6.0–)5.5–6.5–8.0(–9.0) µm (n = 153), wall ca. 0.5 µm. Pycnidia not found.

**Figure 4.**
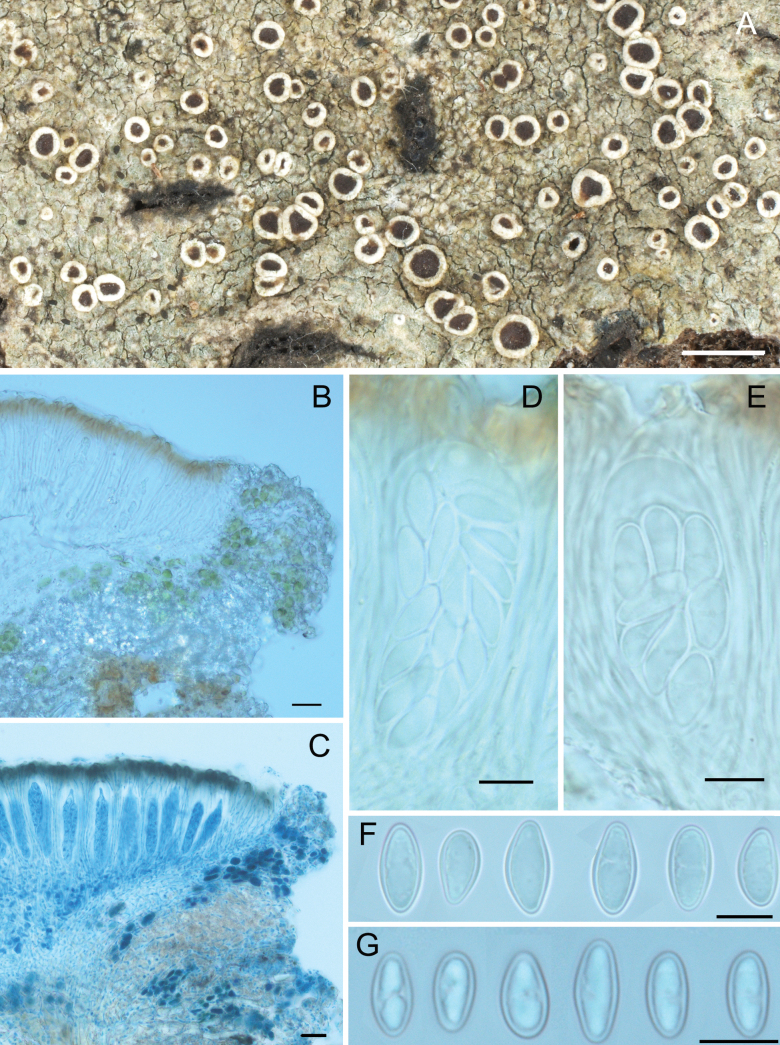
The new species *Lecanorapseudojaponica***A** lichen thallus and apothecia, habit **B** vertical sections of apothecia in polarized light **C** vertical sections of apothecia mounted in lactophenol cotton blue in normal light **D** 16-spored ascus **E** 8-spored ascus **F** ascospores in 8-spored asci **G** ascospores in 16-spored asci. Scale bars: 1 mm (**A**); 20 µm (**B, C**); 10 µm (**D, E, F, G**).

##### Chemistry.

Thallus K+ yellow, C-; containing atranorin, zeorin and the stictic acid complex.

##### Distribution.

This species occurs on bark at similar localities as *L.anhuiensis* in Anhui Province at elevations between 1160 and 1720 m.

##### Etymology.

The specific epithet refers to the similar species *L.japonica*.

##### Notes.

In our collections, we observed the presence of apothecia containing 8-spored asci as well as others containing 16-spored asci, growing mixed and distributed randomly on the same thallus. On rare occasions, 8-spored asci have been also been observed in the apothecia containing 16-spored asci. In order to rule out the possibility that we were actually observing two species growing intermixed, we sequenced both types of apothecia separately. The phylogeny (Fig. 2) showed no genetic differences between these apothecia. The only phenotypic difference seems to be that ascospores in 8-spored asci are, on average, larger than those in 16-spored asci, although both have a similar size range. It might be confused with other species of the *L.subfusca* group, especially if only 8-spored asci are found in the hymenium, but it is readily distinguished by its unique chemistry.

Three multispored species from the *L.subfusca* group previously reported from China, *L.japonica*, *L.subjaponica* and *L.moniliformis*, are morphologically similar to *L.pseudojaponica* in having red-brown apothecia, an epihymenium without crystals and small crystals in the amphithcium. *Lecanorajaponica* differs by the lack of lichen substances other than atranorin (Miyawaki 1988), *L.subjaponica* contains (16–) 32-spored asci and lacks stictic acid (Lü et al. 2012) and *L.moniliformis* has crenate apothecial margins and produces psoromic acid (Qiu and Lü 2022). *Lecanorasubpraesistens* is another multispored species with an egranulose epihymenium, but it can be distinguished from *L.pseudojaponica* by large crystals in the amphithecium, slightly larger apothecia (0.5–1.5 mm) and the absence of stictic acid (Nayaka et al. 2006).

##### Additional specimens examined.

China: Anhui Prov.: Anqing Ci., Yuexi Co., Yangtianwo, Yaoluoping National Nature Reserve, 31°58′11″N, 116°4′10″E, alt. 1160 m, on bark, 15 Oct 2020, Yao Zongting 20200915 (HMAS-L-0147401), Yao Zongting 20200911 (HMAS-L-0147400), Yao Zongting 20200932 (HMAS-L-0147405); Lu’an Ci., Jinzhai Co., the main peak of the Tiantangzhai Scenic Area, Da-pie Mountain, 31°06′20″N, 115°46′15″E, alt. 1720 m, on bark, 12 Oct 2020, Ren Qiang 20200751 (HMAS-L-0147385); Lu′an Ci., Jinzhai Co., Waterfalls area of the Tiantangzhai Scenic Area, 31°12′26″N, 115°76′67″E, alt. 1492 m, on bark, 19 Sep 2022, Zhang Yanyun 22-959 (AHUB-00813); Lu′an Ci., Jinzhai Co., Waterfalls area of the Tiantangzhai Scenic Area, 31°12′27″N, 115°76′69″E, alt. 1490 m, on oak bark, 19 Sep 2022, Zhang Yanyun 22-961 (AHUB-00815); Lu′an Ci., Huoshan Co., Baimajian in the Main Scenic Area of Ta-pieh Mountain, 31°11′45″N, 116°17′95″E, alt. 1459 m, on bark, 09 Sep 2021, Zhang Yanyun 21-124 (AHUB-01027).

##### Material of additional species examined.

*Lecanorabaekdudaeganensis*: China. Anhui Prov.: Anqing Ci., Yuexi Co., 31°10′16″N, 115°35′35″E, alt. 770 m, on bark, 13 Oct 2020, Zhang Jiarong 20200766 L1452 (HMAS-L-0147386), Zhang Jiarong 20200762 (HMAS-L-0147387).

*Lecanoracateilea*: China. Yunnan Prov.: Diqing Tibetan Autonomous Prefecture, Baima Snow Mt., 27°24′00″N, 98°56′99″E, alt. 4100 m, on stump, 23 Oct 2003, Wang Lisong et al. 03-22910 (KUN-L).

*Lecanoraloekoesii*: China. Shaanxi Prov.: Baoji Ci., Taibai Mt., 33°54′20″N, 107°47′99″E, alt. 2200 m, on Betula bark, 2014, Wang Lisong et al. 14-45264 (KUN-L-47212); Heilongjiang Prov.: Heihe Ci., Sunwu Co., 49°38′99″N, 127°17′59″E, alt. 335 m, on bark, 24 Aug 2014, Wei Xinli et al. HLJ201400311 (HMAS-L-0131277); Yichun Ci., Hongxing Co., 49°48′01″N, 127°25′34″E, alt. 280 m, on bark, 26 Aug 2014, Wei Xinli et al. HLJ201400640 (HMAS-L-0131305); Yichun Ci., Fenglin Co., 49°46′01″N, 127°25′20″E, alt. 262 m, on bark, 27 Aug 2014, Wei Xinli et al. HLJ201400853 (HMAS-L-0131321).

*Lecanorasubjaponica*: China. Yunnan Prov.: Diqing Tibetan Autonomous Prefecture, Baima Snow Mt., 27°24′00″N, 98°56′99″E, alt. 4100 m, on bark, 23 Oct 2003, Wang Lisong et al. 03-22905 (KUN-L); Xizang Prov.: Lizhi Ci., 29°43′99″N, 94°43′99″E, alt. 3131 m, on bark, 19 Aug 2004, Huang Manrong 1662 (HMAS-L-0148671).

### ﻿Key to the multispored species of *Lecanora* and similar genera with lecanorine apothecia and multispored asci

**Table:** 

1	Asci *Fuscidea*-type, 32–200-spored	***Maronea* (Fuscideaceae)**
–	Asci *Lecanora*-type or *Biatora*-type	**2**
2	Asci *Biatora*-type, ascospores globose, 4.5–5.5 µm diam., asci 24–32-spored, only known from the type locality in Greenland at an elevation of 20 m	***Lecanorapolysphaeridia***
–	Asci *Lecanora*-type	**3**
3	Ascospores narrowly ellipsoid to fusiform to elongate, asci 8–100-spored	**4**
–	Ascospores ellipsoid, asci 8–32-spored	**5**
4	Paraphyses branched and anastomosing, asci 8–64-spored, containing depsidones	***Neoprotoparmelia* (Parmeliaceae)**
–	Paraphyses slender and mostly simple, asci 32–100-spored, containing depsides	***Maronina* (Parmeliaceae)**
5	Apothecial discs epruinose or occasionally slightly pruinose	**6**
–	Apothecial discs pruinose	**17**
6	Thallus K+ yellow	**7**
–	Thallus K-	**15**
7	Amphithecium with large crystals	**8**
–	Amphithecium with small crystals	**9**
8	Epihymenium without crystals (POL-), with red-brown pigmentation not altered by K, asci 12–16-spored, only known from the type locality in northern India, at elevations between 2500 and 2800 m	***Lecanorasubpraesistens***
–	Epihymenium with crystals (POL+, K-sol)	**10**
9	Epihymenium with fine crystals (POL+, K-sol, N-insol)	**11**
–	Epihymenium without crystals (POL-)	**12**
10	Apothecia sessile, 0.3–0.7 mm diam., discs red-orange, epihymenium yellowish-brown, asci 8–(16)-spored, only known from the type locality in Kenya at elevations between 1500 and 2000 m	***Lecanorapleospora***
–	Apothecia sessile to subimmersed, 0.5–1.6 mm diam., discs red-brown to blackish-orange, epihymenium reddish-brown to yellowish-brown, asci (8–)12–16-spored, known from different parts of the Alps and Ukraine at elevations between 900 and 2000 m	***Lecanorapraesistens***
11	Apothecial discs yellowish-brown, epruinose or slightly pruinose, amphithecium with small crystals (K-sol), asci 16-spored, producing atranorin, zeorin, usnic and norstictic acid, known from China, South Korea and the Russian Far East, at elevations between 150 and 2900 m	***Lecanoraloekoesii***
–	Apothecial discs yellowish-brown to deep brown, amphithecium with small crystals (K-insol), asci 16-spored, producing atranorin and zeorin, known from the east of China at elevations between 850 and 1720 m	***Lecanoraanhuiensis***
12	Asci (16–)32-spored, ascospores 7.5–12.5 × 4.0–6.0 μm, apothecia 0.5–1.6 mm diam., discs shiny, apothecial margin entire, producing zeorin, only known from China at elevations between 2400 and 3800 m	***Lecanorasubjaponica***
–	Asci 8–16 spored, apothecia usually smaller than 1 mm diam	13
13	Apothecial discs plane to convex, margin crenate, asci (8–)12–16-spored, producing atranorin and psoromic acid, only known from China at elevations between 1300 and 1700 m	***Lecanoramoniliformis***
–	Apothecial discs plane to concave, margin entire or slightly flexuous, asci 8- and 16-spored	**14**
14	Apothecia crowded, only with atranorin, known from Japan and China at elevations between 70 and 2700 m	***Lecanorajaponica***
–	Apothecia dispersed to aggregated, with zeorin and stictic acid complex in addition to atranorin, known from China at elevations between 1300 and 1700 m	***Lecanorapseudojaponica***
15	Apothecia disc red brown to black brown, asci 16(–32)-spored, no lichen products, known from Europe and North America	***Polyozosiasambuci***
–	Apothecia disc yellow brown or brown, asci 12–16(–32)-spored, containing usnic acid	**16**
16	Apothecia 0.2–0.5 mm diam., disc yellowish, epruinose, asci 12–16-spored, ascospores simple, with fumarprotocetraric acid besides usnic acid, only known from Yunnan Province in south-western China at elevations of 3500 m	***Lecanorashangrilaensis***
–	Apothecia 0.5–1 mm diam., disc brown, usually slightly pruinose, asci (12–)16(–32)-spored, ascospores simple or 1-septate, with zeorin besides usnic acid, known from north-eastern Spain at elevations between 25 and 700 m	***Lecanorastrobilinoides***
17	Apothecial section P-, discs red brown to dark brown, with slightly to heavily pruinose, asci 12–16-spored, only known from the type locality in north-eastern China at the elevation between 350 and 400 m	***Lecanoraweii***
–	Apothecial section P+ yellow	**18**
18	Apothecia densely clustered, discs yellowish-brown to orange-brown, with heavily whitish-grey pruina, asci (8–)12(–14)-spored, known from Northern Hemisphere	***Lecanoracateilea***
–	Apothecia scattered, discs red brown, with heavily bluish-grey pruina, asci 12–16-spored, known from the type locality in Mexico and China	***Lecanorabruneri***

## Supplementary Material

XML Treatment for Lecanora anhuiensis

XML Treatment for Lecanora pseudojaponica

## ﻿Additional information

### Author ORCIDs

Lijuan Li https://orcid.org/0000-0003-1048-1971

Yanyun Zhang https://orcid.org/0000-0002-0902-5066

Christian Printzen https://orcid.org/0000-0002-0871-0803

### Data availability

All of the data that support the findings of this study are available in the main text.

## References

[B1] Alstrup V (1993) News on lichens and lichenicolous fungi from the Nordic countries. Graphis Scripta 5: 96–104.

[B2] Arup U Ekman S Lindblom L Mattsson JE (1993) High performance thin layer chromatography (HPTLC), an improved technique for screening lichen substances. Lichenologist (London, England) 25(1): 61–71. 10.1006/lich.1993.1018

[B3] Brodo IM (1984) The North American species of the *Lecanorasubfusca* group. Beiheft zur Nova Hedwigia 79: 63–185.

[B4] Brodo IM Haldemann M Malíček J (2019) Notes on species of the *Lecanoraalbella* group (Lecanoraceae) from North America and Europe. The Bryologist 122(3): 430–450. 10.1639/0007-2745-122.3.430

[B5] Culberson CF Kristinsson H (1970) A standardized method for the identification of lichen products. Journal of Chromatography A 46: 85–93. 10.1016/S0021-9673(00)83967-9 5072880

[B6] Davydov EA Yakovchenko LS Hollinger J Bungartz F Parrinello C Printzen C (2021) The new genus Pulvinora (Lecanoraceae) for species of the ‘*Lecanorapringlei*’ group, including the new species *Pulvinorastereothallina*. The Bryologist 124(2): 242–256. 10.1639/0007-2745-124.2.242

[B7] Ferencová Z Rico VJ Hawksworth DL (2017) Extraction of DNA from lichen-forming and lichenicolous fungi: A low-cost fast protocol using Chelex. Lichenologist (London, England) 49(5): 521–525. 10.1017/S0024282917000329

[B8] Gardes M Bruns TD (1993) ITS primers with enhanced specificity for basidiomycetes – application to the identification of mycorrhizae and rusts. Molecular Ecology 2(2): 113–118. 10.1111/j.1365-294X.1993.tb00005.x 8180733

[B9] Giralt M Gómez-Bolea A (1991) *Lecanorastrobilinoides*, a new lichen species from north-eastern Spain. Lichenologist (London, England) 23(2): 107–112. 10.1017/S0024282991000270

[B10] Guderley R Lumbsch HT (1999) Notes on multispored species of *Lecanora* sensu stricto. Lichenologist (London, England) 31(2): 197–210. 10.1006/lich.1998.0190

[B11] Guindon S Dufayard JF Lefort V Anisimova M Hordijk W Gascuel O (2010) New algorithms and methods to estimate maximum-likelihood phylogenies: Assessing the performance of PhyML 3.0. Systematic Biology 59(3): 307–321. 10.1093/sysbio/syq010 20525638

[B12] Han LF Zhao JC Guo SY (2009) *Lecanoraweii*, a new multispored species of *Lecanora* s. str. from northeastern China. Mycotaxon 107(1): 157–161. 10.5248/107.157

[B13] Kalb K (1991) Lichenes Neotropici ausgegeben von Klaus Kalb. Faszikel XII (No. 476–525). Neumarkt OPf.

[B14] Kondratyuk SY Lőkös L Jang SH Hur JS Farkas E (2019) Phylogeny and taxonomy of *Polyozosia*, *Sedelnikovaea* and *Verseghya* of the Lecanoraceae (Lecanorales, lichen-forming Ascomycota). Acta Botanica Hungarica 61(1–2): 137–184. 10.1556/034.61.2019.1-2.9

[B15] Laundon JR (2003) The status of *Lecanorazosterae* in the British Isles. Lichenologist (London, England) 35(2): 97–102. 10.1016/S0024-2829(03)00013-6

[B16] Laundon JR (2003) Six lichens of the *Lecanoravaria* group. Nova Hedwigia 76(1–2): 83–111. 10.1127/0029-5035/2003/0076-0083

[B17] Li LJ Wang LS Printzen C (2023) A new species and new combination of *Lecanora* s. str. (Lecanoraceae) from China. Lichenologist (London, England) 55(3–4): 115–124. 10.1017/S0024282923000142

[B18] Lü L Zhao ZT (2017) *Lecanorashangrilaensis* sp. nov., on pinecones from China. Mycotaxon 132(2): 441–444. 10.5248/132.441

[B19] Lü L Joshi Y Elix JA Lumbsch HT Wang HY Koh YJ Hur JS (2011) New and noteworthy species of lichen genus *Lecanora* (Ascomycota; Lecanoraceae) from South Korea. Lichenologist (London, England) 43(4): 321–329. 10.1017/S0024282911000144

[B20] Lü L Zhang LL Liu XL Zhao ZT Wang HY (2012) *Lecanorasubjaponica*, a new lichen from China. Lichenologist (London, England) 44(4): 465–468. 10.1017/S002428291200014X

[B21] Lumbsch HT (1994) Die *Lecanorasubfusca*–Gruppe in Australasien. The Journal of the Hattori Botanical Laboratory 77: 1–175. 10.18968/jhbl.77.0_1

[B22] Lumbsch HT Plümper M Guderley R Feige GB (1997) The corticolous species of *Lecanora* sensu stricto with pruinose apothecial disks. Acta Universitatis Upsaliensis (Symbolae Botanicae Upsaliensis 32(1): 131–161.

[B23] Mangold A Martín MP Lücking R Lumbsch HT (2008) Molecular phylogeny suggests synonymy of Thelotremataceae within Graphidaceae (Ascomycota: Ostropales). Taxon 57: 476–486. https://www.jstor.org/stable/25066016

[B24] Medeiros ID Mazur E Miadlikowska J Flakus A Rodriguez-Flakus P Pardo-De la Hoz CJ Cieślak E Śliwa L Lutzoni F (2021) Turnover of lecanoroid mycobionts and their *Trebouxia* photobionts along an elevation gradient in bolivia highlights the role of environment in structuring the lichen symbiosis. Frontiers in Microbiology 12: 774839. 10.3389/fmicb.2021.774839 PMC8721194 34987486

[B25] Minh BQ Nguyen MAT von Haeseler A (2013) Ultrafast approximation for phylogenetic bootstrap. Molecular Biology and Evolution 30(5): 1188–1195. 10.1093/molbev/mst024 23418397 PMC3670741

[B26] Miyawaki H (1988) Studies on the *Lecanorasubfusca* group in Japan. The Journal of the Hattori Botanical Laboratory 64: 271–326.

[B27] Miyawaki H (1994) *Lecanoraimshaugii*, a lichen of eastern North America and eastern Asia. The Bryologist 97(4): 409–411. 10.2307/3243907

[B28] Nayaka S Upreti DK Lumbsch HT (2006) Two new *Lecanora* species from India. Lichenologist (London, England) 38(5): 421–424. 10.1017/S0024282906005731

[B29] Øvstedal DO Fryday AM Lewis Smith RI (2020) *Lecanoramuscigena* (Lichenized Ascomycota, Lecanorales), a new lichen species in the *Lecanorafuscescens* group from South Georgia. New Zealand Journal of Botany 58(2): 145–152. 10.1080/0028825X.2019.1682625

[B30] Pérez-Ortega S Kantvilas G (2018) *Lecanorahelmutii*, a new species from the *Lecanorasymmicta* group from Tasmania. Herzogia 31(1(2)): 639–649. 10.13158/heia.31.1.2018.639

[B31] Qiu L Lü L (2022) *Lecanoramoniliformis* sp. nov. from China. Mycotaxon 137(3): 465–469. 10.5248/137.465

[B32] Rambaut A Drummond AJ Xie D Baele G Suchard MA (2018) Posterior summarization in Bayesian phylogenetics using Tracer 1.7. Systematic Biology 67(5): 901–904. 10.1093/sysbio/syy032 29718447 PMC6101584

[B33] Rodriguez Flakus P Printzen C (2014) *Palicella*, a new genus of lichenized fungi and its phylogenetic position within Lecanoraceae. Lichenologist (London, England) 46(4): 535–552. 10.1017/S0024282914000127

[B34] Ronquist F Teslenko M van der Mark P Ayres DL Darling A Höhna S Larget B Liu L Suchard MA Huelsenbeck JP (2012) MrBayes 3.2: Efficient Bayesian phylogenetic inference and model selection across a large model space. Systematic Biology 61(3): 539–542. 10.1093/sysbio/sys029 22357727 PMC3329765

[B35] Śliwa L (2007) A revision of the *Lecanoradispersa* complex in North America. Polish Botanical Journal 52(1): 1–70. https://www.jstor.org/stable/23321028

[B36] Trifinopoulos J Nguyen LT von Haeseler A Minh BQ (2016) W-IQ-TREE: A fast online phylogenetic tool for maximum likelihood analysis. Nucleic Acids Research 44(W1): W232–W235. 10.1093/nar/gkw256 PMC4987875 27084950

[B37] Vilgalys R Hester M (1990) Rapid genetic identification and mapping of enzymatically amplified ribosomal DNA from several *Cryptococcus* species. Journal of Bacteriology 172(8): 4238–4246. 10.1128/jb.172.8.4238-4246.1990 2376561 PMC213247

[B38] Wang CL Sun LY Ren Q Zhao ZT (2007) A preliminary study of multispored *Lecanora* from Mt. Taibai. Mycosystema 26(1): 46–50.

[B39] Wang HY Ge AN Li HM Zhao ZT (2013) Additional information on *Lecanoraloekoesii*. Mycotaxon 123(1): 235–239. 10.5248/123.235

[B40] White TJ Bruns TD Lee S Taylor J (1990) Amplification and direct sequencing of fungal ribosomal DNA genes for phylogenetics. In: Innis MA Gelfand DH Sninsky JJ White TJ (Eds) PCR protocols: a guide to methods and applications.Academic Press, San Diego, 315–321. 10.1016/B978-0-12-372180-8.50042-1

[B41] Zhao X Leavitt SD Zhao ZT Zhang LL Arup U Grube M Pérez-Ortega S Printzen C Śliwa L Kraichak E Divakar PK Crespo A Lumbsch HT (2016) Towards a revised generic classification of lecanoroid lichens (Lecanoraceae, Ascomycota) based on molecular, morphological and chemical evidence. Fungal Diversity 78(1): 293–304. 10.1007/s13225-015-0354-5

